# Pharmacists’ Perspectives on Nicotine Vaping Products (NVPs) for Smoking Cessation in Australia: A Qualitative Analysis

**DOI:** 10.3390/pharmacy13010011

**Published:** 2025-01-29

**Authors:** David Le, Maya Saba, Habib Bhurawala, Muhammad Aziz Rahman, Smita Shah, Bandana Saini

**Affiliations:** 1 Sydney Pharmacy School, Faculty of Medicine and Health, The University of Sydney, Sydney, NSW 2050, Australia; maya.saba@sydney.edu.au (M.S.); bandana.saini@sydney.edu.au (B.S.); 2 The Woolcock Institute of Medical Research, Macquarie University, Sydney, NSW 2113, Australia; smita.shah@health.nsw.gov.au; 3 Paediatric Department, Nepean Hospital, Nepean Blue Mountains Local Health District, Penrith, NSW 2750, Australia; habib.bhurawala@health.nsw.gov.au; 4 Paediatrics, Nepean Clinical School, Faculty of Medicine and Health, The University of Sydney, Sydney, NSW 2050, Australia; 5 Paediatrics, School of Medicine, The University of Notre Dame Australia, Sydney, NSW 2007, Australia; 6 Institute of Health and Wellbeing, Federation University Australia, Berwick, VIC 3806, Australia; ma.rahman@federation.edu.au; 7 Faculty of Public Health, Universitas Airlangga, Surabaya 60115, Indonesia; 8 Prevention Education and Research Unit, Western Sydney Local Health District, North Parramatta, NSW 2151, Australia; 9 School of Public Health, Faculty of Medicine and Health, The University of Sydney, Sydney, NSW 2050, Australia

**Keywords:** health services, nicotine vaping products (NVPs), perceptions, pharmacists, smoking cessation, vaping

## Abstract

Vaping, particularly the use of nicotine vaping products (NVPs), has emerged as a public health concern. The regulatory environment surrounding NVPs in Australia has rapidly evolved, shifting from a prescription-only model to availability through community pharmacies. Pharmacists play a critical role in addressing vaping-related health concerns. This study explores Australian pharmacists’ perspectives on their professional roles and the support needed to manage vaping-related enquiries. Qualitative semi-structured interviews were conducted with 25 practicing pharmacists using a topic guide developed from the current literature and team expertise. The interviews were transcribed verbatim and analysed thematically using an inductive approach to identify key themes. Key themes included *risk perception*, *professional vaping health-related services*, *professional practice* and *other support-related needs*. Pharmacists expressed significant uncertainty about the risks and harms associated with vaping. There was apprehension around the regulatory complexity of supplying NVPs, and participants called for greater education and support, particularly around NVP’s place in smoking cessation and potential vaping cessation services. Effective public health messaging and risk communication about vaping are crucial. At the centre of recent legislative changes, pharmacists require training and professional support to address vaping-related scenarios and provide counselling that aligns with individual risk perceptions, ensuring NVP use is clinically appropriate.

## 1. Introduction

Electronic nicotine delivery systems, known as e-cigarettes, vapes, e-hookahs, vape pens, mods, tanks or vaping products containing nicotine, referred to as simply nicotine vaping products (NVPs), are battery-operated devices that heat a liquid to produce a vapour/aerosol that users inhale in a process described as ‘vaping’. The solutions used in vaping devices may contain high levels of nicotine and a range of chemicals used as flavourings, solvents and liquids required for aerosolization [1]. There is an increased uptake of vaping globally despite the limited evidence about the long-term safety of vaping [2]. A common perception is that vaping a nicotine solution is less harmful than smoking cigarettes, given vaping does not require the combustion of tobacco [3]. However, data compiled from a range of experimental studies indicates that NVP use has a wide range of toxic effects arising from the heat-based decomposition of chemicals in vaping solutions and the material of the vaping device, as well as from inhaling the nicotine-containing aerosol [3]. Nicotine exposure from NVPs has been shown to have an equivalent short-term health impact as conventional cigarettes, for example, on oxidative stress and immune function-mediated inflammatory responses, such as cough and mouth and throat irritation [4]. The long-term effects on respiratory health remain unclear [5]. Another issue is the mislabelling of nicotine content in NVPs, which is well documented across many countries, including in Australia, leaving consumers unknowingly exposed to nicotine content similar to or higher than conventional cigarettes in some cases [6,7].

The status of NVPs as an aid in smoking cessation is an ongoing topic of debate. An ongoing Cochrane review, for example, suggests that while there is evidence that NVPs may increase quit rates compared to conventional nicotine replacement therapies (NRTs), the data remain imprecise with few robustly conducted randomised controlled trials [8]. Most evidence summaries on this issue highlight the need for further research [9]. Regardless, from a clinical perspective, in 2023, Australian guidelines on smoking cessation were revised to include NVPs as a suggested method for trial only in a niche population of highly nicotine-dependent smokers who were unable to quit through conventional NRTs.

**Figure 1:**
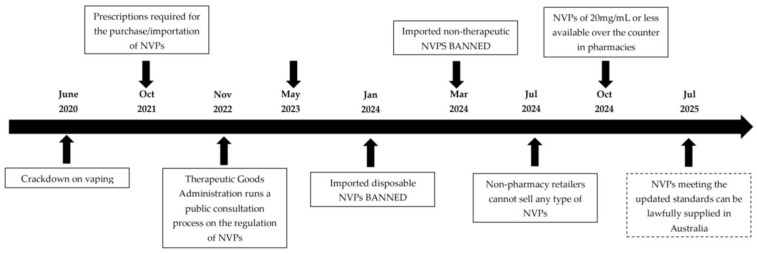
Timeline of Australian Vaping Regulations.

From a public health perspective, concerns have been expressed suggesting that while the ‘therapeutic’ benefits of NVPs in smoking cessation are publicised, the availability of NVPs will likely foster a new generation of nicotine-dependent persons [10]. Indeed, increasing vaping trends have been documented across all Australian age groups, particularly in the 18–24 group, in which a four-fold increase in prevalence from 1.6% (95% CI: 0.8–2.24) in 2019 to 9.3% (95% CI: 7.4–11.2) in 2022–23 is observed [11]. Concerns about the significant public health issues that may be a consequence of these trends, especially in younger adults, have prompted the Australian Federal Government to introduce restrictive legislation to regulate the supply and availability of NVPs (Figure 1) [12]. Initial regulatory changes saw NVPs restricted to a prescription-based supply (October 2021), followed by importation bans on nicotine (January 2024) and then all vaping products (March 2024), as well as the banning of sales of any vapes from any retailer except pharmacies (July 2024). These regulatory moves were based on allowing lawful access to therapeutic NVPs for those medically deemed likely to benefit from their use in terms of smoking cessation [13], whilst restricting any access to NVPs being used for non-clinical reasons [14]. However, in a recent legislative shift, access to NVPs for smoking cessation at a low dose of up to 20 mg/mL is possible for adult consumers from community pharmacies without a prescription [14]. NVPs are now placed in a class of medicines referred to as Schedule 3 in Australia, which requires pharmacist review prior to supply. This current landscape, therefore, imposes a duty of care for Australian pharmacists to ensure the safe supply of NVPs, whether dispensing a prescription or providing them over-the-counter (OTC).

Given that the dispensing or supply of NVPs should occur in line with the Therapeutic Goods Administration (TGA) standards and be subject to state and territory regulations, it is anticipated that pharmacists dispensing NVPs (or supplying NVPs) in a therapeutic paradigm would require them to undertake nicotine dependence assessments, gauge those likely to benefit from using NVPs to support smoking cessation, provide appropriate smoking and vaping cessation counselling, and refer onwards for medical/specialist advice in highly nicotine-dependent patients [15]. While community pharmacists are at the forefront of primary care, it is unclear how Australian pharmacists should or do respond to queries about vaping-related risks and assess and monitor smokers likely to benefit from NVPs as a smoking cessation tool or provide vaping cessation support to those dependent on NVPs. A survey of pharmacy staff in Queensland, Australia, conducted before the 2021 legislative changes allowing the supply of NVPs in pharmacies, indicated that 91% of pharmacy staff felt uninformed and needed training in this area [16].

Other studies exploring health professionals’ views on NVP supply have suggested the need for further training and for structured clinical resources for pharmacists supplying NVPs in the context of smoking cessation [17,18]. Pharmacy-based smoking cessation interventions have enabled trained pharmacists to contribute to a reduction in smoking rates in Australia and globally [19].

This study aimed to explore pharmacists’ clinical awareness, professional support needs, and perceptions of vaping-related supply and counselling services. From 2021 to 2024, whilst these regulatory changes had been rolled out, there were no clinical practice guidelines to assist pharmacists in decision-making or counselling patients about vaping or vaping cessation. These were only very recently developed and published in September 2024. The findings of this study could therefore assist pharmacy educators and professional stakeholders in designing comprehensive training programs to implement practice guidelines for pharmacists.

## 2. Materials and Methods

Ethics approval for this project was obtained from The University of Sydney Human Ethics Committee [2023/748].

To ensure that the quality of the study was as rigorous as possible, the consolidated criteria for reporting qualitative research (COREQ) checklist was adhered to wherever applicable. A detailed description of procedural adherence to this checklist is provided in Appendix A [20].

### 2.1. Study Design

The Theory of Planned Behaviour, which posits that subjective norms, perceived behavioural control and attitudes of people influence professional behaviours, served as a reference for initiating this research [21]. This theory would suggest that the provision of vaping-related health services by pharmacists may depend on their perceptions regarding how peers/colleagues perceive such service delivery (subjective norms) and their own confidence in their capability to deliver such services (perceived behavioural control), as well as attitudes towards vaping. There is robust evidence to suggest that this theory can ‘predict’ health professional behaviours [22], and therefore it was selected to underpin the exploration of pharmacists’ likely vaping service provision behaviours. Pharmacists’ planned behaviours around NVP provision were sought to understand the impact of the vaping regulatory changes [23]. Another theory, the ‘Protection Motivation Theory’, also informed specific lines of query in the interview guide [24]. This theory suggests that for a given ‘risk’ (with vaping services being a risky practice task), people evaluate the likely severity or impact from risk exposure and one’s ability to cope with the risk or have access to effective ‘coping’ strategies [24]. This theory has been utilised in studies on consumers’ vaping/smoking behaviours; hence, it was selected to understand the willingness of pharmacists to engage in practice activity (vaping service provision), which may have been deemed to be ‘risky’ in the current study [25].

### 2.2. Participant Recruitment

Registered Australian pharmacists were invited to participate in semi-structured interviews using a purposive convenience-based and passively snowballed sampling approach. Details of the research project were initially emailed to potential participants who were professional contacts of the research team or those known to researchers as being interested in smoking cessation or respiratory research. Written informed consent was obtained before interviewing participants, and reimbursement for their time was offered as $100 gift vouchers. The recruitment of pharmacists continued until thematic saturation was evident. Themes and codes were generated from data collected from the interviews until a stage where there was no relatively emergent information to inform further analysis [26].

### 2.3. Data Collection

Semi-structured interviews were conducted between February and May 2024 (prior to announcements of NVPs being downregulated to being available in pharmacies without a prescription, i.e., as Schedule 3 medicines). These interviews were conducted virtually via Zoom™ according to the participant’s time preference. An interview guide (Appendix B) was designed based on literature research and the research team’s expertise. The format of the questions was cognitively funnelled, beginning with demographic questions before moving onto in-depth questions that sought to explore the participants’ views on professional roles and practice support needs. Probing questions were also used to capture a complete understanding of the issues discussed. All interviews were audio-recorded, transcribed and then reviewed by the first author.

### 2.4. Data Analysis

Interview transcripts were verified against the audio recordings to ensure accuracy. Each interview was then de-identified and assigned a unique alphabetical code. The interview transcripts were uploaded using NVivo^TM^ 14 software and thematically analysed in an inductive paradigm using Braun and Clarke’s six-step framework for qualitative analyses (Appendix C) [27].

Finally, a subjective analysis was undertaken to identify any differences in thematic derivation between transcripts, based on the participant’s gender, years of experience as a registered pharmacist and pharmacy practice speciality.

## 3. Results

Pharmacists were interviewed until thematic saturation was achieved. Thematic saturation was based on informational redundancy when further interviews revealed no new information [28,29]. This occurred at about the 22nd interview. An amount of 3 further previously confirmed interviews were still conducted, with a total of 25 participants interviewed with no dropouts. The interview duration ranged from 10 to 30 min. Participant demographics and pharmacy characteristics are depicted in Table 1.

**Figure 2:**
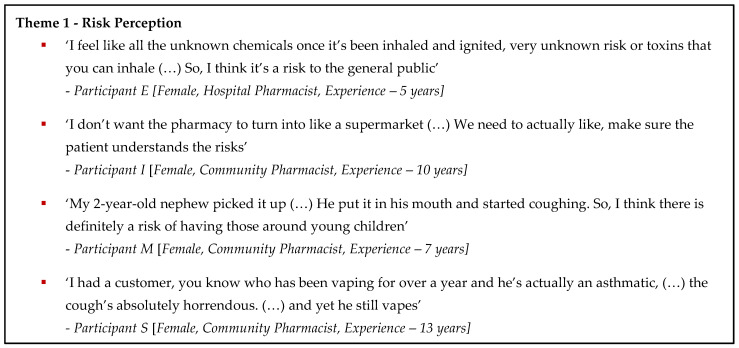

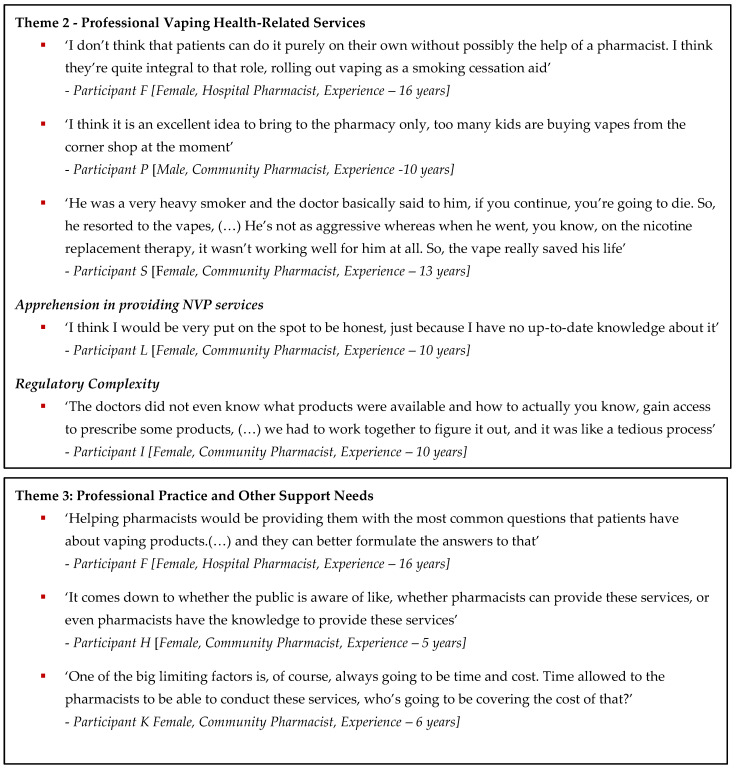
Patient Exemplar Quotes.

Inductive analysis of the data collected during the interviews identified three main themes: (1) Risk Perception, (2) Professional Vaping Health-Related Services and (3) Professional Practice and Other Support Needs. Thematic derivations supported by additional participant exemplar quotes are presented in Figure 2. Most participants reported increasing presentations relating to requests for vaping products or advice in their workplaces.

### 3.1. Theme 1: Risk Perception

Participants agreed that vaping is a risk to individual health and the wider public. Some participants reported this based on observations of consumers who were vaping and who presented to the pharmacy with shortness of breath or other respiratory symptoms. Many participants noted that there appeared to be a perception that vaping nicotine was harmless and safer than smoking cigarettes, especially among adolescents and young adults, who appeared to vape the most. Several participants opined that social media and peer pressure often influenced this.

There were mixed opinions about the comparative safety of NVP use versus cigarette smoking, with some participants believing nicotine vaping was ‘as’ or ‘even more harmful’ compared to cigarette smoking. A common impression of vaping trajectories was that whilst vaping commenced as a choice, it progressed into a habit among users. Some participants described this as a possible ‘gateway effect’ with vaping serving as a precursor to cigarette smoking behaviours. A few participants considered NVPs as a method to combat smoking cessation but acknowledged that the potential risk of nicotine addiction remained. These perceptions were based on participants’ concerns about the uncertainty around the long-term effects of vaping. Participants were also concerned about the risk of second-hand vape exposure.

In addition, participants were unsure about the myriad ingredients within a vaping device, although there were some suggestions that pharmacy/prescription-based NVPs would gain the trust and confidence of consumers. Participants agreed that accessing a pharmaceutical-grade vaping product that has met stringent quality control requirements with accurate labelling of the nicotine and its excipients sounded more trustworthy.

*“I think from a social perspective, society has normalised vaping. But I think that’s also due to the fact that they’ve promoted it and marketed it as something that’s quite harmless”*.

Participant I [Female, Hospital Pharmacist, Experience—10 years]

### 3.2. Theme 2: Professional Vaping Health-Related Services

When asked about opinions around professional vaping-related services, either for the therapeutic provision of NVPs for smoking cessation or supporting the cessation of NVPs, a range of in-depth responses were obtained. Nearly all participants expressed that providing education and information to explain the risks and harms of vaping was a ‘duty of care’ and a ‘harm-reduction’ opportunity for their local community.

There were mixed views on whether NVPs should be used as a smoking cessation aid. Some participants were strongly against recommending NVPs, as they believed that current evidence supported only conventional nicotine replacement therapies, leaving these as the only viable alternative. While several other participants felt there could be a potential place for NVPs in smoking cessation, it would require more evidence. A few other participants also recognised that there may need to be situation-dependent scenarios for NVP provision to support smoking cessation if other avenues had been exhausted. When asked about NVP cessation services, participants suggested pharmacists could potentially assist by recommending lower doses alongside behavioural support.

*“I don’t think it’s a good idea to use vaping as the strategy to quit smoking. But if it’s easier, if it’s a first step for someone, then maybe perhaps it’s a solution. But I don’t think that’s the way to go”*.

Participant H [Female, Community Pharmacist, Experience—5 years]

#### 3.2.1. Subtheme 2.1: Apprehension in Providing NVP Services

When asked about practice experience, only a few participants recalled instances of patients who had come to the pharmacy with NVP prescriptions. Many participants had no prior experience of dispensing NVPs. Participants felt that there was some public demand for NVPs; however, they felt apprehensive about NVP provision, despite the strong notion of having a duty of care to prevent or alleviate harm. This uneasiness appeared to stem from a lack of clinical confidence and clear guidelines to support professional practice.

*“I personally can’t give too much information on it, as I don’t know too much about it”*.

Participant G [Female, Community Pharmacist, Experience—10 years]

#### 3.2.2. Subtheme 2.2: Regulatory Complexity

Apart from a reported uncertainty about the clinical/legal appropriateness of NVP supply, several participants reported being unable to keep up to date given the rapid changes in vaping-related legislation. Some reported that supply chains had not kept pace with regulatory changes; for example, whilst pharmacists had received NVP prescriptions, there were no products currently approved by the TGA that they could order from wholesalers; this necessitated them having to negotiate complex pathways for procurement of NVPs.

Concerning the regulatory policies, most participants supported the imposition of restrictive policies such as upgrading nicotine-containing vaping products to a therapeutic status, where NVPs would require a prescription for use in smoking cessation, rather than consumers sourcing it from illegal or non-pharmaceutical avenues. However, some participants reported difficulty navigating the regulatory framework to supply an NVP on prescription. A few participants reported that the regulatory process to prescribe, dispense and source NVPs was confusing and tedious, where collaboration between doctors and pharmacists had occurred in an attempt to provide a service to a patient requiring an NVP. Despite a willingness to provide smoking/vaping cessation services, the complex nature of the regulation at two levels (national and state/territory) rendered many participants hesitant.

*“Because of like the fact that it’s not approved by the TGA, you need a special authority. So that’s like a bit of a burden”*.

Participant N [Male, Community Pharmacist, Experience—1 year]

### 3.3. Theme 3: Professional Practice and Other Support Needs

All participants expressed an urgent need for education and training. Some participants suggested that workshops and online seminars run by pharmacy organisational bodies would be useful. Others suggested that technical detailing by industry representatives who could explain NVP device usage and discuss potential frameworks for NVP counselling on site would also be effective. Participants’ overall needs appeared to be driven by pragmatism, e.g., how to communicate NVP risks/benefits to patients, how NVP devices operate and dosing and follow-up techniques. There was an expressed need for understanding the place of NVPs in conventional smoking cessation services. Most participants called for practical aids, such as patient education materials, to facilitate effective communication and allow pharmacists to address misconceptions.

Finally, participants were concerned that public perceptions needed to be shaped so that consumers could see pharmacies not just as a point of supply of NVPs but rather as providers of smoking/vaping cessation support. To provide these services viably, some participants raised the issue of remuneration for the time spent, which would acknowledge the training that the pharmacist had undertaken.

*“The public health sector, they should in collaboration with pharmacists…decide how they’re going to tackle this situation…with people who have experience in the community…it needs to be done on an integrative aspect”*.

Participant W [Female, Community Pharmacist, Experience—13 years]

Notably, the generation of codes underpinning the themes did not subjectively differ based on participant attributes (age, gender, experience) in subjective analyses that compared the frequency of codes generated across transcripts. Based on a planned post-analysis reflective debrief by team members, it was acknowledged that as the main data analysts (DL, BS and MS) were all pharmacists, a professional lens may have influenced coding and theme derivation, leading to confirmatory bias.

## 4. Discussion

This study is the first to explore Australian pharmacists’ perspectives on vaping-related health services since regulatory changes to make NVPs a prescription product were implemented. Participants expressed significant concerns about vaping, viewing it as a high-risk behaviour, particularly due to uncertainties about its long-term health effects, the role of NVPs in smoking cessation, and the increasing use of these products among adolescents. Though Australian vaping policies may be different from those of other countries, with restrictions around vape availability only in the context of clinical need, pharmacists worldwide will need to incorporate health services to combat vaping; the results of our study therefore have global relevance.

These results resonate with those reported in a recent opinion poll of the readership of the Australian Journal of Pharmacy, which is a professional journal (n = 1096 respondents, August 2024), where pharmacists indicated that vapes should be taxed and regulated (26%), banned entirely (29%), be prescription items (22%) or available OTC (7%)—reflecting the unease and risk perceptions voiced among our participants [30]. This poll was undertaken immediately after the regulatory position shift to allow pharmacists to supply Schedule 3 or non-prescription supplies for adult NVPs [31]. The willingness to supply NVPs without a prescription appears to be the least favoured option in this poll. Of course, our research was conducted prior to a change in the regulation, and even though ‘prescription-based supply’ appears to have more support, as indicated in the above poll, our participants were speculative even of this option. It is not surprising then that there has been a furore in professional pharmacy circles after the regulatory shift to NVPs being made available without prescription through pharmacies was announced in June 2024 [30]. Several key pharmacy organisations have suggested a lack of consultation by policymakers, leaving pharmacists trying to work out their required roles in an ‘eleventh hour’ regulatory change to allow NVPs as non-prescription pharmacist supply items. There is apprehension that political drivers may have motivated this change [30]. Interestingly, other Australian researchers have reported ‘political interference’ as being a rate-limiting factor in regulatory attempts to curb the uptake of NVPs in Australia [10]. However, some researchers had advocated for the non-prescription availability of NVPs in pharmacies, suggesting that many would likely resort to illicit use, likely to be more harmful, given such products would not be adherent to required quality standards. Our participants also emphasised that pharmaceutical-grade products legislated for pharmacist provision would offer people access to quality-assured products rather than purchasing products from illicit sources, which may be potentially harmful. Although tentatively, health economic modelling has portrayed that less restrictive access may afford higher public health gains [32]. These are valid points favouring non-prescription NVP supply by pharmacists; however, it would appear that pharmacist practitioners may not, in reality, be willing or ready to accept this role [18]. Similarly, public health researchers are also likely to have a different view, given the growing evidence of harm from NVPs and the notion that the downregulation/of NVPs from ‘prescription only’ to ‘non-prescription’ supply by pharmacists may signal to consumers that NVPs are safe [33].

It was evident in our thematic analysis that the introduction of a prescription-only regulatory model for NVPs (i.e., the regulation in place when interviews were conducted) had presented healthcare professionals with challenges. Other research studies have also reported that Australian health professionals find it difficult to grapple with the added burden of navigating the regulatory framework around NVPs. Similar experiences have been reflected in the legalisation of medical abortion drugs in NSW, where there have been varying degrees of uncertainty, complexity and concern expressed by doctors [34]. Likewise, pharmacists displayed an unwillingness to engage with the provision of the emergency contraceptive pill without a prescription, citing concerns about protocol and risk behaviours [35]. At the time of our research, there were no Therapeutic Goods Administration (TGA)-approved NVPs, which necessitated pharmacists researching products. This regulatory complexity packs an additional layer to the uncertainty evidently experienced by our participants around the supply of NVPs. Of course, this has been exacerbated with the unanticipated regulatory shift to allowing non-prescription availability.

The strong perception of risk voiced by our participants aligns with contemporary understanding of factors influencing risk assessment, such as *uncertainty* (e.g., of evidence of benefits versus harms) or *vulnerability* (more uptake by adolescents and young adults), which can negatively mediate risk perceptions [36]. Participants repeatedly expressed uncertainty around evidence for the safety of long-term NVP use or for NVP use in facilitating conventional smoking cessation [37]. Many were concerned about the increased uptake of vaping among adolescents, suggesting that this population would be very vulnerable to long-term health harms associated with vaping. Participants further noted the negative impact of social media platforms on their risk perceptions around vaping, which is an established determinant that can mould risk perceptions [38,39]. Certainly, in a content analysis of Australian pharmacy news sources, authors estimated that the ‘representation’ of vaping was portrayed negatively, with risk representations outweighing benefit representations, which is likely to build negatively influenced heuristics in pharmacist readers [40]. It has been proposed that given the clinical and regulatory reality of NVPs as non-prescription items that will require pharmacist interventions around judicious supply, be it upon prescription or without, accurate relative risk-based information may help pharmacists (and the public) arrive at a realistic decisional balance around providing or not providing NVPs to individuals [41].

The current public health debate and regulatory shifts may also swing the perceptions of current or potential NVP consumers. The Royal Australian College of General Practice (RACGP) has listed NVPs as a last resort for patients attempting to quit smoking after the failure of approved pharmacotherapies. Given this case, some patients who meet the medical criteria for NVP prescription may present to a pharmacy but hold a perception that NVP use is high risk, based on the current public debate, making them reluctant to try this approach. Community pharmacists filling the prescription would need to be able to provide clear benefits versus risks information. On the other hand, some patients request NVPs from pharmacies irrespective of any risks. These situations may place pharmacists in a clinical conundrum that requires skilful handling. Clinical hesitancy, as observed in our data, has also been observed in pharmacists when called upon to supply products to which public debate/controversy is linked, such as naloxone to prevent opioid overdose [42]. In an exploration of pharmacists’ views on supplying naloxone over the counter, clinical hesitancy appeared to be based on under-confidence in clinical knowledge about opioid overdose as well as the impact on businesses where stigma may be attached to those using injectable opioids [42]. This was a clear observation in our data also.

Most participants recognised their duty of care in providing a professional, balanced overview of NVPs to minimise the risks and harms of vaping. Harm minimisation programs have been integral to the professional services that Australian pharmacists offer. Pharmacists have participated in needle exchange services as well as opioid substitution programs, which have benefitted the wider community [43]. Hence, moving forward, it may be useful to pinpoint harm reduction services; this is indeed the approach that has led professional organisations to construct practice guidelines (recently published in September 2024). Across various services, pharmacists have expressed a preference for collaborative service provision with general practitioners/physicians, and again, in advancing the pharmacy-based NVP supply model (prescription-based or non-prescription supply), a collaborative model is recommended [14].

For all healthcare professions, communication is a core skill taught in pre-registration curricula, but ‘risk communication’ is not specifically taught, and there is a paucity of research on how pharmacists undertake this [44]. Many models have been proposed to improve risk communication. The Extended Parallel Process Model (EPPM) is one such model. It suggests that risk perceptions can determine health behaviours, leading to a proactive, protective ‘danger control’ path or an avoidant, less useful ‘fear control’ path [45]. Protective actions are taken when there is a high perception of risk/threat and a firm belief in self-coping skills. Thus, pharmacists may need to gauge the risk stance of patients requesting NVPs and effectively transition patients towards undertaking danger control behaviours using targeted communication [46]. Models such as the EPPM are effective, for example, when used to gauge the responsiveness of public health workers in a potential pandemic through perceived risk communication, in which training programs could be developed to address these attitudes [47]. This can be replicated with pharmacists, where the apprehension observed can be minimised by structured training programs on risk communication training to enable confidence and the ability to assess risk in a vaping health-related service.

It was clear from our data that pharmacist participants strongly expressed a need for clinical guidelines, which have only recently been made available [48]. The next step in this timeline would be to facilitate pharmacists to translate the guidelines, which are couched from a harm minimisation approach, into a deliverable health service. For example, such service implementation training should outline the structures (S) and processes (P) required to provide the service and outcomes (O) necessary to demonstrate ongoing service provision to patients, where the SPO model is considered the framework for defining the quality of health services [49]. While participants suggested some structures and processes, it would be difficult to undertake without professional support and remuneration [50]. An end-to-end service involving nicotine addiction assessment, product selection, counselling, follow-up and referral/triage with specialist services would require time management, in addition to investment in resources such as staff training, space allocation (counselling room area and product shelf space) and rostering additional staff to cover pharmacist dispensing duties as depicted in Table 2. Remuneration for pharmacists providing these services is therefore important either via user-paid or health system-funded pathways. This will require practice research testing pharmacist-provided NVP supply in the context of smoking cessation as well as vaping cessation services for clinical impact and cost-effectiveness.

### Strengths and Limitations

While the purposive convenience snowballing approach may have introduced a sampling bias, efforts were made to ensure participant diversity by recruiting pharmacists from various practice settings and with different experience levels to ensure a maximally varied sample. To further mitigate confirmation and researcher bias, regular peer debriefing sessions were held with the research team. Although participant validation was not feasible due to the participants’ time constraints, methodological triangulation was employed by comparing the interview findings with the existing literature and related data sources to enhance the credibility of the results. The results may not be generalisable to all pharmacists; nonetheless, this robustly conducted qualitative study offers valuable insights into the experiences of a specific group of pharmacists, which may resonate with or inform similar contexts and stimulate future research in this important area. Survey instruments can now be designed to collect generalisable data from nationally representative samples of the pharmacist population, following the results of this study.

## 5. Conclusions

Vaping presents significant concerns, particularly among adolescents and young adults. Within an evolving regulatory landscape, Australian pharmacists are key to managing vaping-related risks. This study highlights their uncertainty, hesitancy and lack of confidence in supplying NVPs for smoking cessation. Hence, effective public health messaging and risk communication about vaping are crucial. Pharmacists, being the most accessible primary healthcare professionals, require comprehensive training to address vaping-related scenarios and provide clinical counselling that aligns with individual risk perceptions, ensuring NVP use is clinically appropriate.

## Figures and Tables

**Table 1:** Participant Demographics and Pharmacy Characteristics.

Demographic Variables	Sample, *n* (*n* = 25) (%)
Gender	
Female	22 (88)
Male	3 (12)
Pharmacy Background	
Community	16 (64)
Hospital	9 (36)
Experience as a registered pharmacist (Years)	
<1–5	4 (16)
6–10	13 (52)
11–15	6 (24)
>16	2 (8)
Pharmacy Qualification	
BPharm	20 (80)
MPharm	5 (20)
Additional Study *	5 (20)
**Pharmacy Characteristics**	
Average number of prescriptions dispensed each day	
<50	1 (4)
51–100	3 (12)
101–250	12 (48)
251–400	4(16)
>401	5 (20)
Number of pharmacy staff on an average day	
1–5	4 (16)
6–10	10 (40)
11–19	7 (28)
>20	4 (16)
Type of pharmacy participants work in	
Banner Group	8 (32)
Independent	8 (32)
Private Hospital	6 (24)
Public Hospital	3 (12)
Pharmacy provides Smoking Cessation Services	
Yes	25 (100)
Consult Area for General Enquiries/Professional Services	
Yes	21 (84)
No	4 (16)
*Average Smoking Cessation Consults by participants (Weekly)*	
0–4	12 (48)
5–10	3 (12)
Unsure	10 (40)
*Experience with Dispensing Vaping Products*	
Yes	6 (24)
No	19 (76)

* BPharm: Bachelor of Pharmacy; MPharm: Master of Pharmacy. HMR: Home Medicines Review Accredited Pharmacist; Graduate Certificate of Pharmacy Practice; MPhil: Master of Philosophy; PhD: Doctorate of Philosophy; Graduate Diploma of Clinical Pharmacy.

**Table 2:** Handling of NVP requests and needs for service support.

Handling of a Patient/Consumer Request for a Vaping Product	Resources/Support Needs to Offer Vaping-Related Smoking Cessation Services
**STRUCTURE** ▪ Counselling Room ▪ Adequate Staffing ▪ Stock Maintenance of NVPs ▪ HONC/Fagerstrom Assessments ▪ Guidelines ▪ Placebo Devices ▪ Pharmacy Staff Training ▪ Pharmacist Training	**TRAINING (ALL)** ▪ **Specific Training Topics** • Counselling/Nicotine addiction behaviours (Some) • Training specific to products (ALL) • Communication skills (Some) ▪ **Training Formats** • Online (ALL) • Modules (ALL) • Hands-on (Most) • On the job (Some) **RESOURCES** ▪ Public risk/health messaging (Most) ▪ Public/consumer messaging on evidence for vaping as a smoking cessation method (Few) ▪ Specialised vaping cessation clinics/Quitline (Very Few) ▪ NVP product information for patients (Some) **SUPPORT (ALL)** ▪ Local health district/PHN involvement with training (Some) ▪ Government/Organisational bodies (ALL) ▪ More GP training (Few) ▪ Interprofessional collaboration (Very Few) ▪ Public education campaigns (Most) ▪ Industry-sponsored programs for pharmacists (Some) ▪ Remuneration for vaping/smoking cessation services (Some)
**PROCESS** ▪ Information Gathering Phase • Smoking History • Nicotine Addiction • NRT Use • Quit Attempts ▪ Action • Proactive support in GP referral • Referral to regular GP • Pros and Cons of vaping as smoking cessation discussed • Comprehensive consult • Counselling on device use Information provision	
**OUTCOME** ▪ Recommendation: Vaping prescription products are recommended only if prior attempts with NRT have been unsuccessful ▪ Documentation of outcome ▪ Follow-up support: Nicotine addiction review/success with smoking cessation ▪ Referral to other support services [Quitline]	

NVPs: Nicotine Vaping Products. HONC: Hooked on Nicotine Checklist. NRT: Nicotine Replacement Therapy. GP: General Practitioner. PHN: Primary Health Network.

**Figure A1:**
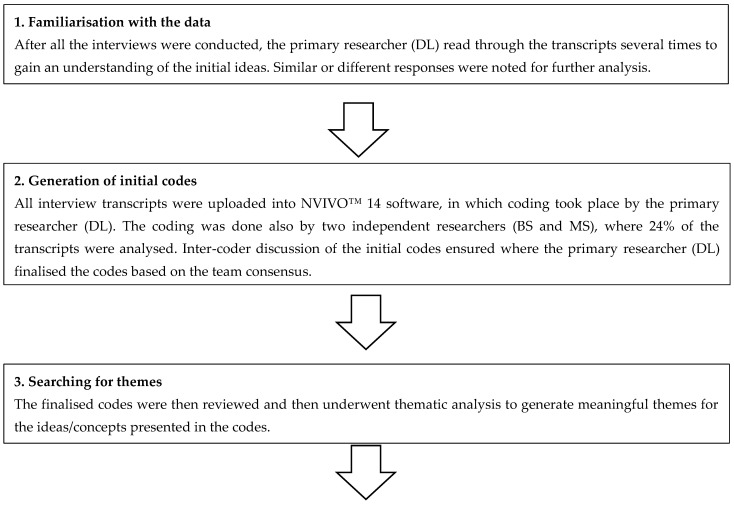

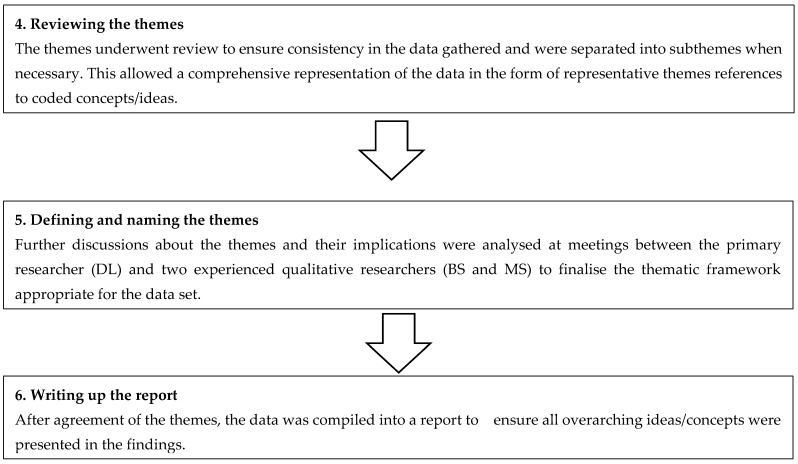
The Multi-Phased Thematic Analysis Process.

## Data Availability

The original contributions presented in this study are included in the article. Further inquiries can be directed to the corresponding author(s).

## Appendix A

**Table A1:** COREQ Checklist.

Topic	Item Number	Researcher Report and reference to pages item described in the main manuscript.
**Domain 1: Research Team and Reflexivity**		
*Personal Characteristics*		
Interviewer/Facilitator	1	Mr David Le (DL) was the person who conducted the interviews and was the primary researcher for this study. Page 4.
Credentials	2	DL holds a Bachelors of Pharmacy and a Master of Public Health degree.
Occupation	3	DL is also a registered pharmacist, currently practicing as a hospital pharmacist as well as being enrolled as a Higher Degree Research student completing a Master of Philosophy.
Gender	4	Male.
Experience and training	5	Trained on interview skills, coding and theming primarily provided by a senior qualitative researcher, Professor Bandana Saini (BS), with support from Dr Maya Saba (MS). Others authors with prior experience in qualitative research e.g., Dr Habib Bhurawala/Prof Smita Shah and Prof Muhammad Aziz Rahman also provided relevant direction and training.
*Relationship with Participants*		
Relationship established	6	The seed participants were approached based on being professional contacts of DL or BS or those who had been previous research participants and expressed a desire for invitations to future research to BS. Other participants were snowballed from original contacts and researchers had no established relationship with these latter participants.
Participant knowledge of the interviewer	7	Participants were likely alerted about the credentials of the research team through the participant information sheet that was emailed to potential participants as per the approved ethics protocol. Page 4.
Interviewer characteristics	8	DL has qualifications in pharmacy and public health with an interest in harm reduction roles in pharmacy, given his Master of Public Health. DL is also an early career pharmacist with 10 years of experience as a pharmacist as well as 5 years of experience as a sessional academic tutoring pharmacy students at the University of Sydney.
**Domain 2: Study Design**		
*Theoretical Framework*		
Methodological orientation and Theory	9	As seen in Study Design. Page 4.
*Participant Selection*		
Sampling	10	A purposive convenience snowball sampling method was employed.
Method of approach	11	Potential participants were emailed an invitation on a professional email address known through them being 1) professional contacts of the research team (DL and BS) or 2) participants of prior respiratory research who had consented to be contacted about future research (BS).
Sample size	12	Twenty-five registered pharmacists.
Non-participation	13	None.
*Setting*		
Setting of data collection	14	Online, i.e., interviews were conducted on Zoom, a videoconferencing platform (Zoom.us).
Presence of non-participants	15	None.
Description of sample	16	As seen in Table 1 *Participant Demographics and Pharmacy Characteristics.* Pages 5–6.
*Data Collection*		
Interview guide	17	As seen in Appendix B.
Repeat interviews	18	None.
Audio/visual recording	19	Only the audio recordings were downloaded from Zoom™ onto a password protected laptop and then stored on the University’s Research Data Storage to protect against data breaches.
Field notes	20	Field notes were taken where appropriate to aid in grasping the ‘full picture’ in data analysis. These notes were referred to during data analysis for clarification or for any nuances that may have been missed.
Duration	21	Interviews ranged from 10–30 min as highlighted in the Section 3 of the manuscript. Page 5.
Data saturation	22	As seen in Results. Pages 4–5.
Transcripts returned	23	No, this was a limitation of the study.
**Domain 3: Analysis and Findings**		
*Data Analysis*		
Number of data coders	24	There were three data coders DL, BS and MS. Of the transcripts 10% were coded by all three—BS MS, and DL. Team debriefs ensued to agree to a coding structure and then DL coded the remainder of the transcripts.
Description of the coding tree	25	**A summary of the coding tree** **Views on Vaping** - Risk/Harm to Youth - Evidence in smoking cessation - Lack of Education/Awareness - Impact on Health - Apprehension **Pharmacist Roles** - Counselling and Advice - Smoking Cessation - Vaping Cessation - Regulation **Support and Education Needed ** - Resources and Training - Guidelines and Protocols - Patient Stimulated Scenarios
Derivation of themes	26	The research team reviewed transcripts and there were regular debriefs with the primary and senior researchers. DL and BS debriefed multiple times during the data analysis and thematic derivation process. MS an independent researcher also reviewed the data and assisted with the thematic derivation process. Discussion of the results of the analysis between the research team and using a consensus approach (DL, BS, SS and MS).
Software	27	NVivo™ 14 software as seen in the Section 2.4 of the manuscript. Page 4.
Participant checking	28	Participants did not provide feedback on the findings, but some have opted to receive the results of the study once completed.
*Reporting*		
Quotations presented	29	As seen in Figure 2 *Patient Exemplar Quotes.* Pages 6-7.
Data and findings consistent	30	Yes, as seen in the Section 3 of the manuscript. Page 9.
Clarity of major themes	31	Yes, as seen in the Section 3 of the manuscript. Pages 7–9.
Clarity of minor themes	32	Yes, as seen in the Section 3 of the manuscript. Pages 8–9.

## Appendix B

**Table A2:** Semi-Structured Interview Guide for Pharmacists.

▪ **Interview Questions**	**Prompts to be Used Only if Needed**	**Aim of the Question**
**First, I would like to ask you about your background as a pharmacist. This is so we can see differing opinions between pharmacists** - *What is the length in years of your total experience as a registered pharmacist?* - *Please would you tell me about your HIGHEST pharmacy qualification?* - *Was this degree completed in Australia?* - *How would you describe the pharmacy where you primarily work?* - *How many staff would there be in your pharmacy during regular opening hours?* - *Approximately how many prescriptions would your pharmacy dispense on a regular day?*	▪ Not applicable	This question aims to establish participant characteristics and pharmacy demographics
▪ **I would like to ask about the smoking cessation services your pharmacy provides. Would you describe them for me?**	▪ Pharmacy area dedicated to this/visible displays? ▪ Consult area options for smoking cessation ▪ Any staff trained in smoking cessation ▪ Average smoking cessation consults in a week? ▪ Most common methods used by staff to offer smoking cessation (motivation/NRT/Rx) ▪ Any collaborative experience with other healthcare professionals in the area	This question aims to determine the level/pattern/experience of smoking cessation by the pharmacist within their workplace
▪ **In general, what are your opinions on vaping/vaping products?**	▪ Vaping as a habit or choice ▪ Relative risk of smoking versus vaping ▪ Impact of vaping on health/public health ▪ Vaping products and ingredients	Vaping is a recent phenomenon which has caused debate and controversy. This question seeks to understand how perspectives on vaping have been shaped.
*Consider a patient who comes into your pharmacy; they are known to you as an ex-smoker. They ask you about your advice regarding vaping. How would you respond? Would you have different advice if this was a young adult?*	▪ Not applicable	This question aims to understand how pharmacists would manage a vaping-related health request.
▪ **Finally, I would like to ask you about any practice support resources you feel pharmacists would need in order to implement vaping cessation services?**	▪ Training (formats/courses/topics) ▪ Practice guidelines ▪ Support with specific services patients can be referred to ▪ Resource materials for patient education	This question aims to elicit what pharmacists would feel to be confident in providing vaping-related health services.
